# Symptoms of Anxiety and Depression and Sense of Coherence in Patients Undergoing Carotid Artery Stenting

**DOI:** 10.3390/ijerph191912222

**Published:** 2022-09-27

**Authors:** Magdalena Piegza, Izabela Jaworska, Kamil Bujak, Paweł Dębski, Łukasz Kunert, Karina Badura-Brzoza, Maciej Żerdziński, Michał Błachut, Jacek Piegza

**Affiliations:** 1 Department of Psychiatry, Faculty of Medical Sciences in Zabrze, Medical University of Silesia in Katowice, 42-612 Tarnowskie Gory, Poland; 2 Department of Cardiac, Vascular and Endovascular Surgery and Transplantology, Silesian Center for Heart Diseases, Medical University of Silesia in Katowice, 41-800 Zabrze, Poland; 3 Third Department of Cardiology, Faculty of Medical Sciences in Zabrze, Silesian Center for Heart Diseases, Medical University of Silesia in Katowice, 41-800 Zabrze, Poland; 4 Dr. Krzysztof Czuma’s Psychiatric Center, Psychiatric Department No 2, 40-340 Katowice, Poland

**Keywords:** anxiety, depression, carotid artery stenting, sense of coherence, social support, carotid atherosclerosis

## Abstract

Background: The sense of coherence is lower in patients with somatic diseases and psychiatric disorders. Purpose: The purpose of this study was to evaluate the intensity of depression and anxiety symptoms and their relationship with the sense of coherence and to try to determine the relationship between the strength of sense of coherence and symptoms of depression and anxiety with the presence of symptoms of carotid atherosclerosis in a group of patients undergoing carotid artery stenting. Methods: 35 patients, including 13 with symptomatic atherosclerosis in the carotid arteries, completed self-report tests: Hospital Scale of Depression and Anxiety (HADS) and the SOC-29 Life Orientation Questionnaire (SOC-29), 22 of whom also rated their subjective feelings of anxiety and depression on a scale included in the author’s questionnaire. Results: Both symptomatic and asymptomatic patients did not differ significantly in the severity of depression, but they differed in anxiety levels as assessed by the HADS scale. There were no differences in the overall strength of sense of coherence and its individual components. Nearly 12% of those undergoing carotid artery stenting have pronounced anxiety symptoms, and just over 14% have pronounced depression symptoms. A higher overall sense of coherence and its components are associated with lower severity of depression symptoms. Lower severity of anxiety correlates negatively with a higher sense of understanding, meaningfulness, and holistic Sense of Coherence (SOC). Manageability appeared higher in men. Conclusions: SOC is an important health-promoting factor that is preferably related to mental health parameters of patients with carotid atherosclerosis.

## 1. Introduction

Depressive and anxiety disorders often accompany cardiovascular disease. Most data are available on ischemic heart disease and heart failure [1,2,3,4,5,6]. Relatively little information is available on the symptoms of depression and anxiety in patients with carotid atherosclerosis and their relationship with the sense of coherence (SOC) [7,8,9].

The authors of the study decided to examine the relationships between the above parameters and tried to determine whether the presence of carotid atherosclerosis symptoms differentiates the group examined. The idea to examine patients with carotid atherosclerosis constitutes a consequence of the project previously carried out regarding the assessment of depression and anxiety symptoms and the sense of coherence in women who have undergone coronary arteriography. Stemming from that study, women with cardiac syndrome X appeared to experience a higher level of anxiety several months after coronary angiography compared to women with diagnosed significant atherosclerosis in coronary vessels [4]. There were no statistically significant differences in the estimation of depressive symptoms in both groups [5]. Moreover, women with no significant stenosis of coronary arteries demonstrated a lower sense of comprehensibility before and after coronarography and lowered manageability after the procedure [6]. Therefore, the idea was conceived to examine whether some similar relations could be observed in the group of patients with lesions in carotic arteries, dividing these individuals into the groups of symptomatic and asymptomatic patients.

More pieces of research concern the comorbidity of emotional disorders in patients after cerebral incidents, showing that comorbidity, particularly the coexistence of several psychiatric diagnoses, is associated with more frequent rehospitalizations or death within 6 months after stroke [10,11]. In light of the increasing need for consultative psychiatry, the search for health-promoting psychological factors as a kind of protection against the potentially traumatizing effects of illness on patients’ mental functioning remains an important issue. One of the approaches within which the perspective of health psychology is being developed is salutogenesis. Salutogenesis is a psychological approach to the issue of health and illness that competes with pathogenesis, emphasizing the need to seek and strengthen preventive and health-promoting factors in the human psyche. According to the concept of salutogenesis, proposed by Aaron Antonovsky, coherence consists of the sense of comprehensibility, manageability, and meaningfulness [12,13]. All three elements constitute the overall sense of coherence and play slightly different roles in the process of maintaining health. Coherence is one of the most well-known and documented psychological factors of salutogenetic importance. The sense of coherence is lower in the case of people with somatic diseases and psychiatric disorders. It can also be an important factor in how people cope with illness [14,15]. People with higher SOC cope better with stressful situations, identify stimuli as less threatening, and enjoy good health longer. SOC is a predictor of future health and is a valuable aspect of health promotion [14]. In addition, a high sense of coherence is associated with a better quality of life and a lower intensity of depression and anxiety symptoms in patients with various psychiatric disorders [16] and is a favorable factor in the course of some somatic diseases [17].

Purpose: The purpose of this study was to assess the intensity of depression and anxiety symptoms in a group of patients undergoing carotid artery stenting and their relationship to the sense of coherence, and also to determine the differences in the strength of the sense of coherence and symptoms of depression and anxiety, taking into consideration the symptoms of carotid atherosclerosis (symptomatic and asymptomatic patients). For this purpose, symptoms of depression and anxiety, as well as the sense of coherence before stent implantation in a Zabrze center, were assessed. This study is part of a larger project that has been approved by the Bioethics Committee of the Silesian Medical University, ref. no. NN-6501-132/07.

## 2. Material and Methods

Of the initial 47 patients of the cardiology department of the Silesian Center for Heart Diseases in Zabrze who were qualified for carotid artery stenting in three consecutive years, only 35 completed the Hospital Scale of Depression and Anxiety (HADS) and the SOC-29 Life Orientation Questionnaire. Only 22 patients determined subjective feelings of depression and anxiety. The study included both symptomatic (13 patients) and asymptomatic (22 patients) patients, who formed Group 1 (Gr 1) and Group 2 (Gr 2), respectively. Patients were qualified for revascularization based on their clinical history and the degree of internal carotid artery stenosis, which was ≥50% for symptomatic patients and ≥70% for asymptomatic patients (Doppler USG). The history of ischemic stroke and/or transient ischemia (TIA) in the area supplying the narrowed artery within the last six months prior to the study was used as a symptomatic criterion. The inclusion criteria covered, in addition to consent to participate in the study and eligibility for carotid artery angioplasty with stent implantation, the age over 18 years, and the absence of cognitive impairment to the extent that tests could not be performed.

## 3. Instruments

The Polish adaptation of the ***SOC-29 Life Orientation Questionnaire*** by Aaron Antonovsky was used to assess the sense of coherence. It consists of 29 questions allowing one to estimate on a point scale the value of the overall sense of coherence (SOC-TOTAL) and its three components: the sense of comprehensibility, manageability, and meaningfulness. Numerous studies show that reaching a value of about 140–160 points indicates a strong sense of coherence, 110–130—medium, and below 100—a low sense of coherence [6,15].

***The Hospital Anxiety and Depression Scale*** (HADS) used in our study consists of 14 questions, respectively; 7 related to symptoms of anxiety (HADS A subscale) and 7 to symptoms of depression (HADS D subscale). A maximum of 3 points can be obtained for each question. Higher scores indicate greater severity of depression and anxiety symptoms. The scale can be used both to screen for emotional disorders and to assess the severity of the symptoms. The cut-off point is considered to be 7 points, with scores from 8–10 indicating borderline symptoms and 11–21 points indicating marked severity [18,19].

As in other publications, patients rated their level of depression and anxiety on a scale of 0 to 10, indicating a subjective sense of the presence of symptoms commonly categorized by psychiatrists as depression and anxiety symptoms, which is not equivalent to an objective diagnosis [6,20]. This evaluation included a retrospective period covering the time before being informed of the need to undergo carotid artery stenting. In addition, a proprietary questionnaire was used. It included questions concerning socio-demographic data and selected clinical parameters.

## 4. Statistical Analysis

All the statistical analyses were performed using Statistica version 13.3 (TIBCO Software, Palo Alto, CA, USA) and R (R Core Team (2022)). R is a language and environment for statistical computing. (R Foundation for Statistical Computing, Vienna, Austria). Categorical data are shown as the number of patients and percentages. Quantitative variables are presented as mean (standard deviation; SD) if normally distributed or median (interquartile range; IQR). The normality of the distribution of continuous variables was tested by the Shapiro–Wilk test. Quantitative variables were compared using an independent samples *t*-test or Mann–Whitney U test, as appropriate. The Cochran–Armitage test was applied to assess trends in binomial proportions across the levels of a single variable. Spearman’s rank correlation coefficient was utilized to investigate the relationship between two numerical variables. Finally, multiple linear regression models with HADS A and HADS D as dependent variables were performed. The level of statistical significance was set to α < 0.05 (two-tailed).

## 5. Results

Ultimately, 35 people participated in the study, 65.7% of whom were men. The demographic characteristics of the entire study group are shown in Table 1.

Both symptomatic (Gr 1) and asymptomatic (Gr 2) patients did not differ significantly from each other in the severity of depression, similarly to the case of SOC and its components. However, the difference has been observed in anxiety as assessed on the HADS scale (Table 2). Most of those undergoing carotid artery stenting showed the absence of significant depression and anxiety symptoms on this scale (Table 3).

Due to the small number of patients in both groups, marital status, source of income, and presence of somatic diseases were not compared when taking into consideration the results of the tests used. The size of the groups made it possible to compare the patients in terms of the analyzed parameters, taking into account gender. There were no significant differences between men and women except for manageability which was higher in men (mean = 49.0 (SD = 9.8) vs. 57.3 (11.3); *p* = 0.03). It was observed that there was no correlation of SOC parameters with the declared presence of stressful situations, and symptomatic and asymptomatic patients did not differ in the values of individual components of the sense of coherence and the total score. Therefore, the analysis of the studied parameters (depression symptoms, anxiety, sense of coherence) was performed for the whole group of people participating in the study.

Patients with a higher sense of meaningfulness scored significantly lower on the depression subscale of the HADS (HADS D) (r = −0.35; *p* = 0.04). The same correlation was noted for manageability and the HADS D scale (r = −0.49; *p* = 0.003). The strongest negative correlation (r = −0.62, *p* < 0.001) was found between the understanding component (comprehensibility) of the sense of coherence and depression symptoms and the holistic sense of coherence (SOC) and depression on the HADS D scale (r = −0.62, *p* < 0.001). Similarly, people with higher comprehensibility and higher overall SOC scale scores presented lower levels of anxiety on the HADS A subscale (r = −0.44; *p* = 0.009, r = −0.40; *p* = 0.02, respectively). The sense of meaningfulness, as the only component of SOC, was found to have a significant relationship with the level of anxiety obtained on the 0–10 point scale (r = −0.43; *p* = 0.045), with a near borderline value for the manageability (r = −0.35; *p* = 0.058). The correlation of overall SOC score with anxiety level was negative (r = −0.45; *p* = 0.04). There were no significant correlations of the analyzed SOC parameters with the sense of depression assessed on the 0–10 point scale. The correlations have been presented in Table 4.

## 6. Discussion

The available literature shows that little attention has so far been paid to the issue of emotional distress in the context of patients with carotid atherosclerosis, as opposed to the coronary atherosclerosis population. The situation is even worse with the sense of coherence, for which the keywords ‘sense of coherence’ and ‘carotid artery stenting’ did not yield a single result (PubMed database) while entering the keywords ‘sense of coherence’ and ‘carotid atherosclerosis’ yielded only two articles neither of which is cited in this study.

The results of our study indicate that almost 12% of those undergoing carotid artery stenting have pronounced symptoms of anxiety, and just over 14% have pronounced symptoms of depression. Such patients require psychiatric consultation and probably psychological support. To the best of our knowledge from other centers, post-stroke patients burdened with psychiatric disorders are predisposed to repeat hospitalizations or adverse events, including death, compared to post-stroke patients without accompanying psychiatric disorders. This is particularly true for depression and anxiety syndromes, with post-stroke patients with anxiety disorders more likely to die within six months of discharge from a rehabilitation ward [11].

In a retrospective cross-sectional study of more than 37,000 U.S. stroke patients undergoing carotid artery stenting (CAS) or carotid endarterectomy (CEA), 18.5% had concomitant psychiatric disorders or diseases. Patients with diagnoses of psychosis, depressive disorders, substance abuse, and several comorbid psychiatric diagnoses benefited less from revascularization procedures than patients without a psychiatric history. It is worth noting that this finding applies to men, as this relationship was not observed in women [10].

In our study, there were no significant differences in the intensity of depression between symptomatic and asymptomatic patients, as well as in SOC scores. However, a difference in anxiety level was observed. In contrast to our previously completed project concerning women undergoing coronary stenting, where we showed differences in the components of SOC, women without significant coronary artery stenosis had lower comprehensibility and lower manageability compared to women with significant coronary atherosclerosis. In addition, the lack of support, more frequent use of medical advice, tendency to experience depressive and anxious states, as well as poorer education were associated with lower SOC in both groups of women [6].

The lack of correlation of SOC with the declared experience of stressful situations in our patients is somehow surprising, as there are studies documenting this relationship or focusing on the protective role of support during the experience of stress, which is associated with higher SOC [9,21,22,23,24]. The lack of emotional support also turned out to be an important factor in the development of coronary atherosclerosis [25]. SOC and the sense of experienced support were factors positively related to long-term adherence to secondary prevention of myocardial infarction recommendations and significantly increased the likelihood of the prevention score predicted survival over a 23-year follow-up (1SD increase) [21]. Psychosocial factors, including the sense of coherence and support, had a direct impact on health-promoting behavior and modifiable risk factors for cardiovascular disease. The indirect influence of these factors on pro-health behavior has not been confirmed in the cross-sectional study of the Brazilian population [9].

Other researchers also emphasize that people with ischemic heart disease with low baseline SOC measured before coronary angiography were less willing to modify their habits and lifestyle towards pro-health behaviors compared to patients with high SOC and, therefore, require special attention [26].

The most important dependencies in our study concerned the relationship between the overall sense of coherence and all its components with the intensity of depression symptoms and the overall sense of coherence, comprehensibility, and meaningfulness with the intensity of anxiety symptoms. Correlation analysis showed that the sense of coherence, both globally and taken into consideration as components, is negatively correlated with the elevated parameters of the mental state mentioned above. Similar trends have been described by other researchers [6,8,21,27,28]. The sense of meaningfulness is considered the main component of SOC, responsible for emotional and motivational involvement in creating one’s own life [12]. Many authors see the relationship between lower values, especially of this component, and worse determinants of the mental state. In this study, comprehensibility turned out to be most strongly associated with lower intensity of depression symptoms, as well as the overall sense of coherence. This observation was also confirmed by the prepared regression models, which showed that comprehensibility turned out to be the only factor among the coherence components that may significantly reduce the severity of anxiety and depression symptoms. This may be due to the fact that respondents perceive their environment as more orderly, less threatening, and predictable enough to feel safe enough not to develop symptoms of depression. Moreover, this cognitive component plays a major role in the hierarchy of importance of the SOC components. Similar reasoning can be applied to anxiety symptoms, but the sense of meaningfulness seems to significantly determine the way of coping with stress and constitutes an important protective factor. Among cardiac patients, people with lower levels of depression and anxiety symptoms showed a stronger sense of coherence. One-fifth of the adult population of outpatients with congenital heart disease experienced clinically pronounced anxiety symptoms (based on the self-reported questionnaire) [29].

Neuvonen et al., in their material prepared within the CAIDE (Cardiovascular Risk Factors, Aging, and Dementia) study, confirmed earlier reports on the association of anxiety symptoms with impaired cognitive functioning and an increased risk of Mild Cognitive Impairment/Dementia. Higher levels of anxiety also predicted increased mortality in long-term study participants. On the other hand, a higher sense of coherence played a protective role and was associated with lower mortality in the described group. Depressive symptoms weakened these relationships. The researchers used STAI (State-Trait Anxiety Inventory) to estimate the intensity of anxiety and the Sense of Coherence Scale to measure the sense of coherence [30].

Adolescents with congenital heart disease (median age = 16.3 years) with a strong and time-stable sense of coherence show better adaptation compared to the subgroup of patients with low and/or decreasing SOC. The latter is potentially more vulnerable to adverse clinical outcomes, and the implementation of early psychosocial interventions could improve the quality of functioning of these patients. It should be noted that in this study, SOC (SOC−13) was measured 4 times at 9-month intervals, while patient-reported outcomes (PROs), significant from the point of view of future clinical complications related to the course of the disease, were assumed 27 months after the start of the project that is, at the fourth time point [31]. Similar conclusions were drawn by Swedish researchers who, based on the SOC score in patients after their first myocardial infarction, believe that on this basis, it is possible to identify people who need additional support. The data obtained by them show that women achieved lower SOC scores than men; moreover, people with a high sense of coherence are more physically active, drink more alcohol, experience less stenocardial pain, better understand the essence of the disease, and are more satisfied with the treatment [32].

In another study, high SOC was associated with a lower level of depression, which, according to the authors, emphasizes the usefulness of the assumptions of salutogenesis as an effective strategy in the fight against depression symptoms in patients with risk factors for cardiovascular diseases [6].

The psychometric properties of the Sense of Coherence Scale (SOC-13) were examined in people with risk factors for cardiovascular diseases of primary health care in Majorca, and the adequacy and reliability of the indicators were demonstrated. This variant is suitable for the described population of patients [7].

## 7. Limitations of the Study

The main limitation of the study is the small number of symptomatic and asymptomatic patients undergoing CAS, although a larger population of patients is rarely mentioned in the available literature. In the future, we plan to increase the size of the study population and extend the methodology to include objective tests for the assessment of mental state. The next step will be to assess the stability of the sense of coherence over time and to look for other protective determinants of mental health in people with cardiovascular diseases.

## 8. Conclusions

1. A higher overall sense of coherence and its components is associated with a lower intensity of depression symptoms.

2. A higher comprehensibility, meaningfulness, and higher overall SOC strength are associated with less anxiety.

3. Both symptomatic and asymptomatic patients do not differ in the intensity of depression or in the general strength of the sense of coherence and its individual components, but anxiety levels are higher in symptomatic patients.

4. Men are more manageable.

5. SOC is an important pro-health factor that is associated with mental health parameters of patients with carotid atherosclerosis.

## Figures and Tables

**Table 1:** Socio-demographic and clinical characteristics.

Characteristics	All Patients (*n* = 35)
*Sex* (male)	23 (65.7%)
*Age* (years)	63.4 (10.3%)
*Level of education*	
Primary	7 (20.6%)
Vocational	4 (11.8%)
Secondary	15 (44.1%)
Higher	8 (23.5%)
*Employment*	
Unemployed	1 (2.9%)
Professionally active	3 (8.6%)
Pensioner	31 (88.6%)
*Marital status*	
Single	3 (8.6%)
Married	27 (77.1%)
Widowed	5 (14.3%)
*Previous CAS*	1 (3.0%)
*Previous CAD*	31 (88.6%)
*Previous PCI*	15 (45.5%)
*Previous CABG*	4 (12.1%)
*Malignancy*	3 (8.6%)

Note: Categorical variables are shown as a number of patients (percentage). Quantitative variables are presented as mean (SD). CAS—Carotid Artery Stenting, CAD—Coronary Artery Disease, PCI—Percutaneous Coronary Interventions, CABG—Coronary Artery Bypass Grafting.

**Table 2:** The comparison of SOC components and anxiety and depression scales between groups stratified by the presence of symptoms of carotid artery disease.

Variables	Symptomatic (*n* = 13)	Asymptomatic (*n* = 22)	*p*-Value
COM	49.2 (14.0)	54.4 (11.3)	0.24
MAN	51.5 (9.7)	52.0 (11.8)	0.90
MEA	41.9 (7.4)	40.4 (7.4)	0.55
SOC-TOTAL	142.7 (26.7)	146.8 (26.2)	0.66
HADS A	8 (5–9)	4 (3–8)	0.01
Anxiety Level	4 (2–5)	3 (1–5)	0.62
HADS D	5 (4–7)	3 (1–7)	0.22
Depressiveness	4 (2–5)	2 (2–5)	0.34

Note: COM—comprehensibility, MAN—manageability, MEA—meaningfulness, SOC-TOTAL—sense of coherence total score, HADS A—level of anxiety on the HADS scale, Anxiety Level—level of anxiety on the scale 0–10 points, HADS D—level of depression on the HADS scale, Depressiveness—level of depression on the scale 0–10 points. Components of the SOC scale were distributed normally and are presented as mean (SD). Scales of anxiety and depression were not distributed normally and are therefore shown as median (IQR).

**Table 3:** Distribution of the severity of anxiety and depression symptoms according to the HADS scale in several groups.

	All (*n* = 35)	Symptomatic (*n* = 13)	Asymptomatic (*n* = 22)	*p*-Value *
**HADS A**				0.06
0–7	22 (62.9%)	6 (46.2%)	16 (72.7%)	
8–10	9 (25.7%)	4 (30.8%)	5 (22.7%)	
11–21	4 (11.4%)	3 (23.1%)	1 (4.6%)	
**HADS D**				0.79
0–7	28 (80.0%)	10 (76.9%)	18 (81.8%)	
8–10	2 (5.7%)	1 (7.7%)	1 (4.6%)	
11–21	5 (14.3%)	2 (15.4%)	3 (13.6%)	

Note: * *p*-value for trend (Cochran–Armitage trend test). HADS A—level of anxiety on the HADS scale, HADS D—level of depression on the HADS scale.

**Table 4:** Spearman rank correlation matrix.

	COM	MAN	MEA	SOC-TOTAL	Anxiety Level	HADS A	Depressiveness	HADS D
**COM**	1.00	0.71 ***	0.43 **	0.89 ***	−0.35	−0.44 **	−0.21	−0.62 ***
**MAN**		1.00	0.57 ***	0.90 ***	−0.41	−0.30	−0.20	−0.49 **
**MEA**			1.00	0.69 ***	−0.43 *	−0.16	−0.31	−0.35 *
**SOC-TOTAL**				1.00	−0.45 *	−0.40 *	−0.30	−0.62 ***
**Anxiety Level**					1.00	0.40	0.90 ***	0.44 *
**HADS A**						1.00	0.25	0.39 *
**Depressiveness**							1.00	0.35
**HADS D**								1.00

Note: * *p*-value < 0.05; ** *p*-value < 0.01; *** *p*-value < 0.001; COM—comprehensibility, MAN—manageability, MEA—meaningfulness, SOC-TOTAL—sense of coherence total score, HADS A—level of anxiety on the HADS scale, Anxiety Level—level of anxiety on the scale 0–10 points, HADS D—level of depression on the HADS scale, Depressiveness—level of depression on the scale 0–10 points. Multiple regression models were prepared assuming the influence of coherence components on the severity of anxiety and depression symptoms. A statistically significant factor that could be significant for the decrease in the intensity of HADS A (B = −0.126; *p* = 0.045) and HADS D (B = −0.177; *p* = 0.009) was comprehensibility. Regression models are presented in Table 5.

**Table 5:** Multiple linear regression analysis with HADS A and HADS D as dependent variables.

Depended Variable	Independent Variables	B	Std. Err.	Beta	t	*p*-Value	Adj. R^2^
**HADS A**							0.153
	**COM**	−0.126	0.060	−0.466	−2.093	0.045	
	**MAN**	0.003	0.078	0.009	0.034	0.973	
	**MEA**	−0.020	0.089	−0.043	−0.221	0.826	
**HADS D**							0.308
	**COM**	−0.177	0.064	−0.562	−2.789	0.009	
	**MAN**	0.050	0.082	0.139	0.610	0.546	
	**MEA**	−0.146	0.094	−0.272	−1.552	0.131	

Note: COM—comprehensibility, MAN—manageability, MEA—meaningfulness, HADS A—level of anxiety on the HADS scale, HADS D—level of depression on the HADS scale.

## Data Availability

Data presented in this study are available on reasonable request from the corresponding author.
